# Identification of the Sex Pheromone of the Pink Grass Worm, *Tmetolophota atristriga,* Reveals Possible Population Differences in Male Response to Sex Pheromone

**DOI:** 10.1007/s10886-022-01381-3

**Published:** 2022-09-23

**Authors:** Ashraf M. El-Sayed, Lee-Anne M. Manning

**Affiliations:** The New Zealand Institute for Plant and Food Research Limited, Canterbury Research Centre, Lincoln, 8152 New Zealand

**Keywords:** New Zealand, Noctuidae, *Tmetolophota atristriga*, Sex pheromone, Monitoring, Pasture pest

## Abstract

The pink grass worm, *Tmetolophota atristriga* (Walker), is an endemic New Zealand noctuid moth species that is abundant throughout the North and South Islands. The larvae are minor defoliators of agricultural pasture. We investigated the sex pheromone of this species. Analysis of extract of the female sex pheromone gland identified six compounds: two monounsaturated compounds, (*Z*)-11-hexadecenal (Z11-16:Ald) and (*Z*)-11-hexadecenyl acetate (Z11-16:Ac), three saturated compounds, hexadecanal (16:Ald), hexadecyl acetate (16:Ac) and octadecan-1-ol (18:OH), and a triene hydrocarbon, (3Z,6Z,9Z)-tricosatriene (Z3Z6Z9-23:Hy). Several field-trapping experiments testing combinations of the six compounds were conducted. Results suggested that males of two different populations of *T. atristriga* responded differently to different blends of the compounds. Males of one population responded equally to a two-component blend as to other blends, including the one with all six compounds. By contrast, males of the second population responded only to the six-component blend or a ternary blend of Z11-16:Ald, Z11-16:Ac and Z3Z6Z9-23:Hy. In experiments testing different doses of Z11-16:Ald and Z11-16:Ac in a binary or a six-component blend, a 1 mg dose of the binary blend gave the greatest male catch for both populations. This is the second sex pheromone identification of a New Zealand species of Noctuidae and is the first reported occurrence of Z3Z6Z9-23:Hy as a sex pheromone component of any noctuid species.

## Introduction

The pink grass worm, *Tmetolophota atristriga* (Walker), is an endemic New Zealand noctuid moth species that is abundant throughout the North and South Islands. The larvae are defoliators of agricultural pasture and are currently considered a minor pest. However, changes in climate and farming practices could result in a change in this insect’s pest status. Sex pheromones are used to monitor and control insect pests in agricultural crops and offer many advantages over the use of toxic pesticides, being species-specific, non-toxic and leaving almost no residue. Identification of the sex pheromone for *T. atristriga* could lead to the development of lures to follow the spread of this pest and monitor pest populations during future outbreaks.

New Zealand has a relatively small number of species of Noctuidae, with about 140 endemic species known (Dugdale 1989). A sex pheromone has been identified for only one endemic New Zealand noctuid species, *Graphania mutans* (Walker) (Frérot and Foster 1991). In that study, two distinct taxa within *G. mutans* were found: females from an Auckland population produced (*Z*)-9-tetradecenol (Z9-14:OH), (*Z*)-9-tetradecenyl acetate (Z9-14:Ac), (*Z*)-7-tetradecenol (Z7-14:OH) and (*Z*)-7-tetradecenyl acetate (Z7-14:Ac), while females from a Canterbury population produced these four compounds plus (*Z*)-9-tetradecenal (Z9-14:Ald). Males from each population responded only to the pheromone blend produced by females of the same population.

We therefore undertook to identify the sex pheromone of *T. atristriga* and to develop a lure for monitoring and possible control of this pest. In addition, the identification of the sex pheromone of further New Zealand noctuid species could help shed light on the evolution and speciation of Noctuidae species in New Zealand. Here, we report the identification of the sex pheromone blend of *T. atristriga* and its evaluation in field bioassays in Canterbury, New Zealand.

## Materials and Methods

### Insects

Adult moths were collected by light trapping in Canterbury during the summer months of 2013. All Noctuidae moths were housed individually once caught and transported to the laboratory for identification according to the taxonomic key provided in Dugdale 1988 and Bejakovich and Dugdale 1997. Female and male *T. atristriga* from these samples were maintained individually under a natural summer light and temperature regime.

### Pheromone Gland Extraction

The sex pheromone glands of calling females (n = 5) were dissected during the first 2 h of the scotophase and extracted for 5–10 min in 20 μl of *n*-hexane (Merck Ltd, Darmstadt, Germany) contained within a liquid-nitrogen-cooled 0.5 ml conical vial (Wheaton, Millville, NJ, USA). After all glands had been excised, the vial and its contents were brought to room temperature, and the liquid phase transferred to a 1.1 ml conical glass vial (Alltech, Deerfield, IL, USA) for storage at − 80 °C prior to analysis. Females that were collected from two locations in Canterbury (Lincoln and Little River) were analyzed separately. The sex pheromone glands of 25 females were analyzed from each location. Quantification of each compound in the female sex pheromone gland was conducted using an external standard method (Scott 1995). For this, known amounts (0.1, 1.0, 10.0, and 100.0 ng) of each compound were injected into a gas chromatograph/mass spectrometer (GC/MS). The integrated results were subjected to linear regression analysis (SAS Institute Inc 1998) to determine the relationship between amount and peak area.

### Chemicals

All compounds used as authentic standards in the chromatographic analysis or the field trapping experiments were > 98% chemically and > 99.5% isomerically pure by GC analysis, and were stored at − 80 °C until used. (*Z*)-11-Hexadecenal (Z11-16:Ald), (*E*)-11-hexadecenal, (*Z*)-11-hexadecenyl acetate (Z11-16:Ac), (*E*)-11-hexadecenyl acetate, hexadecanal (16:Ald), hexadecyl acetate (16:Ac) and octadecanol (18:OH) were purchased from Plant Research International, Wageningen, The Netherlands. (3*Z*,6*Z*,9*Z*)-Tricosa- 3,6,9-triene (Z3Z6Z9-23:Hy) was synthesized according to the method described by Gibb et al. (2007).

### Gas Chromatography/Electroantennogram Detector (GC/EAD)

Coupled GC/EAD analysis of pheromone gland extracts were conducted on a Varian 3800 GC equipped with a flame ionization detector (FID), a splitless injector, a 30 m × 0.25 mm (ID) × 0.25 µm VF5-MS capillary column (Factor Four, Varian Inc.) and a Y splitter (Alltech, Deerfield, IL). The column oven temperature was programmed from 80 °C (held for 1 min) to 240 °C at 10 °C.min^−1^. Helium was the carrier gas. The column effluent was split 1:1 between the FID and EAD apparatus. Antennal depolarization was detected using a high resistance EAD probe (Signal Interface Box, Type ID-02) and Intelligent Data Acquisition Controller (Type IDAC-02) (Syntech, Hilversum, The Netherlands). Antennae from 2–3 d-old males collected from Lincoln and Little River were excised at the base and attached to silver electrodes housed in saline-filled glass electrodes using a micromanipulator (Narishige, Tokyo, Japan). Up to five antennal preparations from each location were tested with different female extracts from the same location in GC/EAD analysis.

### GC/MS Analysis

Gland extracts and synthetic chemicals were analyzed on a Saturn 2200 GC/MS (Varian Walnut Creek, CA, USA). The ionization voltage was 70 eV (electron impact ionization) with mass scanning from *m/z* 30–650. The GC/MS was equipped with two different capillary columns: a non-polar 30 m × 0.25 mm ID × 0.5 µm VF5-MS capillary column (Factor four, Varian Inc., USA) and a polar 30 m × 0.25 mm ID × 0.5 µm VF23-MS capillary column (Factor Four, Varian Inc.). For both columns, injection was splitless and the column oven was programmed from 80 °C (held for 1 min) to 240 °C at 10 °C/min^−1^ and then held for 13 min. Compounds were identified by comparing retention times and mass spectra with those of synthetic compounds on the two different columns.

### Dimethyldisulfide (DMDS) Derivatizations

We followed the procedure described by Buser et al. (1983) and Leonhardt and DeVilbiss (1985). Approximately 50 μl DMDS and 5 μl iodine solution (60 mg of I_2_ in 1 ml of diethyl ether) were added to 20 female equivalents in a 1.8 ml glass vial, sealed with a Teflon-lined cap, and held at 40 °C for 15 h. The reaction was quenched with 50 μl of 5% aqueous sodium thiosulphate, and the organic layer dried with anhydrous sodium sulphate before being transferred to a clean 1.5 ml tapered-bottom vial and concentrated with a stream of argon to ca. 10 μl. A 1 μl aliquot (ca. two female equivalents) was analyzed by GC/MS.

### Field-Trapping Experiments

In all field trials, green unitrap bucket traps (International Pheromone Systems Ltd., Cheshire, UK) were suspended 1–1.5 m above the pasture, in a randomized block design, with a minimum of 20 m between traps and 20 m between replicates. Each treatment was randomly assigned to a trapping station within each trapping row. Each trap contained a 2-cm killing strip of dog flea collar (Bayer, Germany), which contained 5% Diazinon insecticide. All the blends (in 150 µl of *n*-hexane) were applied to the large ‘wells’ of red rubber septa (West Pharmaceutical Services, Kearney, NE, USA). The solvent was allowed to evaporate in a fume hood and the septa were stored in heat-sealed foil bags at − 20 °C until use. Pheromone-impregnated septa were placed in the top compartment of a trap. In all field trials, five replicates for each treatment were tested. Traps were checked weekly, and moths were taken to the laboratory for identification (Bejakovich and Dugdale 1997; Dugdale 1988).

#### Individual Compounds

The relative attractiveness of individual EAD-active compounds (Z11-16:Ald; Z11-16:Ac, 16:Ald, 16:Ac,18:OH and Z3Z6Z9-23:Hy) found in the sex pheromone gland were tested in an organic apple orchard near Lincoln, Canterbury, New Zealand. The orchard had a thick, mixed-pasture understorey. Field testing was conducted for three weeks in January 2014 in Lincoln and for three weeks in January 2015 in Little River. Compound loading was 1 mg per septa. Traps baited with a blank lure were used as controls.

#### Binary Blends with Different Ratios of Z11-16:Ald and Z11-16:Ac

The relative attractiveness of three blends with different ratios (75:25, 50:50 and 25:75.) of Z11-16:Ald and Z11-16:Ac were tested in the same organic apple orchard used in the first trial, for three weeks in February 2014. The total loading of the two compounds for all blends was 1 mg. Traps baited with a blank lure were used as controls.

#### Dose–Response of Binary Blend

Three doses (0.1, 1, 10 mg) of the optimum binary blend from the previous trial were tested for catches of male *T. atristriga* in the same organic apple orchard. The trial was deployed for three weeks in April 2014. Traps with a blank lure were used as a control.

#### Minor Compounds Identified in the Sex Pheromone Gland

In a subsequent experiment, four blends were tested to investigate the effect of minor compounds: 1) a two-component blend of Z11-16:Ald and Z11-16:Ac at 0.25:0.75 mg; 2) a five-component blend of Z11-16:Ald, Z11-16:Ac,16:Ald, 16:Ac and 18:OH at 0.25:0.75 0.05:0.05:0.05 mg; 3) a three-component blend of Z11-16:Ald, Z11-16:Ac and Z3Z6Z9-23:Hy at 0.25:0.75:0.05 mg; 4) a six-component blend of Z11-16:Ald, Z11-16:Ac,16:Ald, 16:Ac, 18:OH, and 3Z6Z9-23:Hy at 0.25:0.75:0.05:0.05:0.05 mg. Traps with a blank lure were used as controls. This trial was run for three weeks from January–February 2015 in two locations: a) the same apple orchard as used in the first trial and b) a mixed fruit orchard of cherries, apricots, peaches, plums and nectarines in Little River, Canterbury, New Zealand. The distance between the two sites is ca. 50 km. The experimental design and protocol were identical to the other experiments.

#### Dose–Response of Six-Component Blend

Three doses (0.1, 1, 10 mg) of the six-component blend were tested in the mixed fruit orchard in Little River, Canterbury, New Zealand. The trial was run for three weeks from February–March 2015. Traps with a blank lure were used as controls.

### Data Analysis

The variances of mean captures of each treatment were stabilized using √ (*x* + 1) transformation. Significance of treatments in the field-trapping experiments were tested using ANOVA (SAS Institute Inc 1998). Differences among means were tested using Fisher’s Protected Least Significant Difference.

## Results

### GC/EAD Analysis

**Fig. 1:**
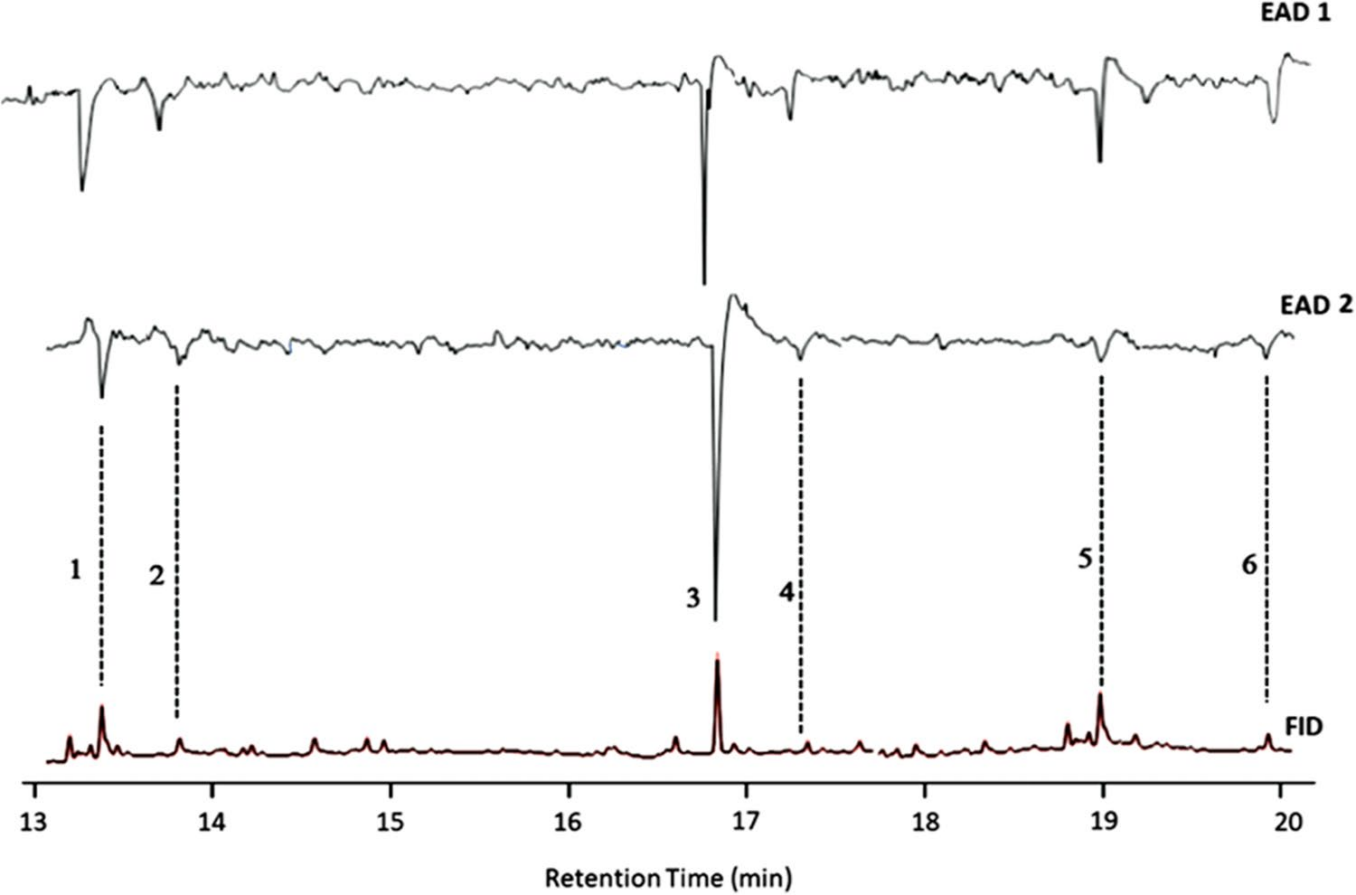
Coupled Gas Chromatography/Electroantennogram Detector (GC/EAD) responses of the antennae of male *Tmetolophota atristriga* to female gland extract. EAD 1 (Little River population), EAD 2 (Lincoln population). A non-polar VF5-MS capillary column was used for the analysis. 1) (*Z*)-11-hexadecenal, 2) hexadecanal, 3) (*Z*)-11-hexadecenyl acetate, 4) hexadecyl acetate, 5) octadecanal, 6) (3*Z*,6*Z*,9*Z*)-Tricosa- 3,6,9-triene

Analysis of female sex pheromone gland extracts by GC/EAD revealed six compounds that consistently elicited EAD responses from male moth antennae (Fig. 1). The GC/EAD profile from the Lincoln population was similar to that of the Little River population (Fig. 1). These compounds were later identified as Z11-16:Ald (1), 16:Ald (2), Z11-16:Ac (3), 16:Ac (4), 18:OH (5) and Z3Z6Z9-23Hy (6). Z11-16:Ac and Z11-16Ald elicited the strongest EAD responses, while the other four compounds elicited more moderate responses.

### Chemical Identification

GC/MS analysis of the EAD-active compounds suggested they were a mixture of saturated and unsaturated aldehyde, acetate, alcohol and hydrocarbons. Comparison of retention times of the EAD-active compounds with synthetic standards on a non-polar column enabled their tentative identification as Z11-16:Ald, 16:Ald, Z11-16:Ac, 16:Ac, 18:OH and Z3Z6Z9-23:Hy. GC/MS analysis of the DMDS reaction showed an adduct with a molecular ion at *m/z* 332 (13%) and diagnostic *m/z* 117 (63%, C_6_H_13_S^+^) and *m/z* 215 (100% C_12_H_23_OS^+^), indicating the addition of DMDS to a double bond at position 11 for Z11-16:Ald. In addition, another adduct had a molecular ion at *m/z* 376 (11%), and diagnostic *m/z* 117 (57%, C_6_H_13_S^+^) and *m/z* 259 (100% C_14_H_27_OS^+^), indicating the addition of DMDS to a double bond at position 11 for Z11-16:Ac. The geometries of the double bonds of these two unsaturated compounds were confirmed by retention time comparisons with synthetic *E* and *Z* isomers of both compounds. The mass spectrum of compound 6 was very similar to the data in Millar (2000) and El-Sayed et al (2013). Further confirmation of the identities of the compounds was confirmed by comparing retention times of authentic standards with gland extract on a polar capillary column. The chemical composition (and amounts) of the sex pheromone gland from females collected from Lincoln was similar to that of females collected from Little River (Table 1).

**Table 1:** Ratio of the six candidate pheromone compounds in the sex pheromone gland extracts of female *Tmetolophota atristriga* from Lincoln and Little River

Compound	Lincoln^a^		Little River^a^	
	ng ± SE/female^a^	Ratio (%)	ng ± SE/female^a^	Ratio (%)
*Z*11-16:Ald	1.2 ± 0.28	26	1.5 ± 0.26	28
16:Ald	0.64 ± 0.16	14	0.84 ± 0.31	16
*Z*11-16:Ac	4.6 ± 1.1	100	5.2 ± 0.84	100
16:Ac	0.86 ± 0.26	19	1.2 ± 0.35	22
18:OH	1.1 ± 0.31	24	1.1 ± 0.35	21
*Z*3*Z*6*Z*9-23Hy	0.94 ± 0.29	21	1.1 ± 0.26	20

^a^ *n* = 25

### Individual Compounds

When the six EAD-active compounds were tested individually at 1 mg loading, none attracted male *T. atristriga*, either at Lincoln or Little River (data not shown).

### Binary Blend with Various Ratios of Z11-16:Ald and Z11-16:Ac

**Fig. 2:**
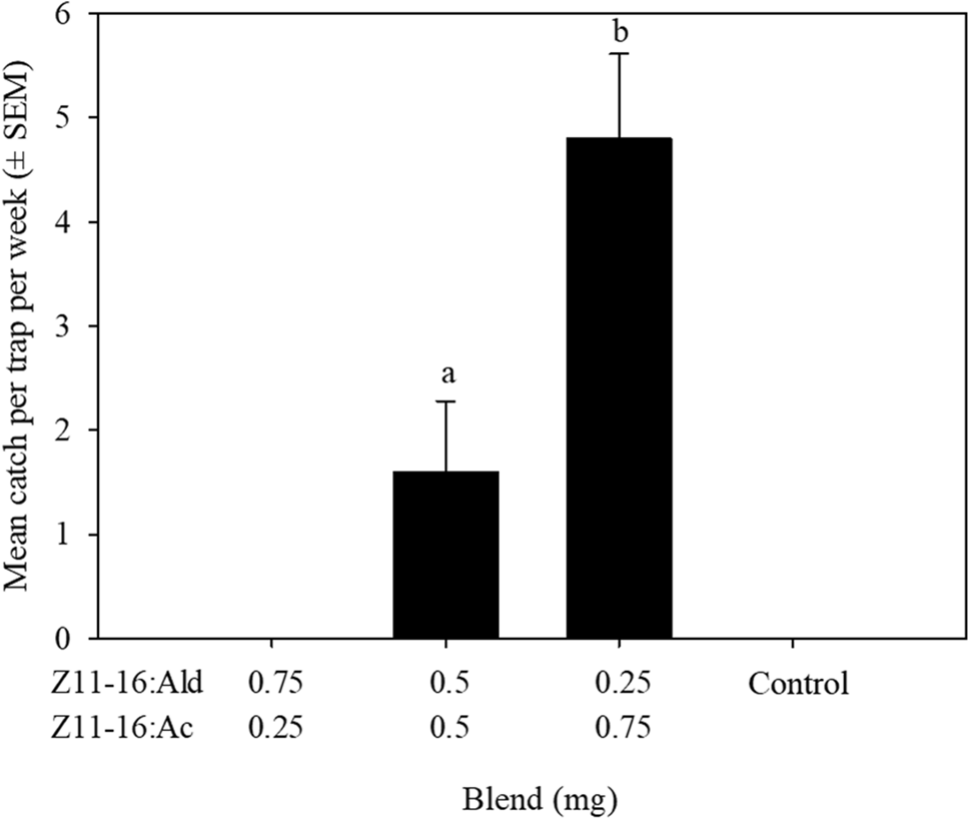
Mean catch ± SEM of male *Tmetolophota atristriga* in traps baited with binary blends containing different ratios of (*Z*)-11-hexadecenal (Z11-16:Ald) and (*Z*)-11-hexadecenyl acetate (Z11-16:Ac)*.* Different letters above columns indicate significant differences (*P* < 0.05)

Changing the ratio of Z11-16:Ald: Z11-16:Ac affected the number of *T. atristriga* caught in traps (Treatment, F_1,8_ = 5.7, *P* < 0.04); Fig. 2). A greater number of male *T. atristriga* were caught in traps baited with a ratio of 0.25:0.75 mg than in traps baited with the 0.5:0.5 mg ratio. No males were caught in traps baited with a ratio of 0.75:0.25 mg (Fig. 2).

### Dose–Response of Binary Blend

**Fig. 3:**
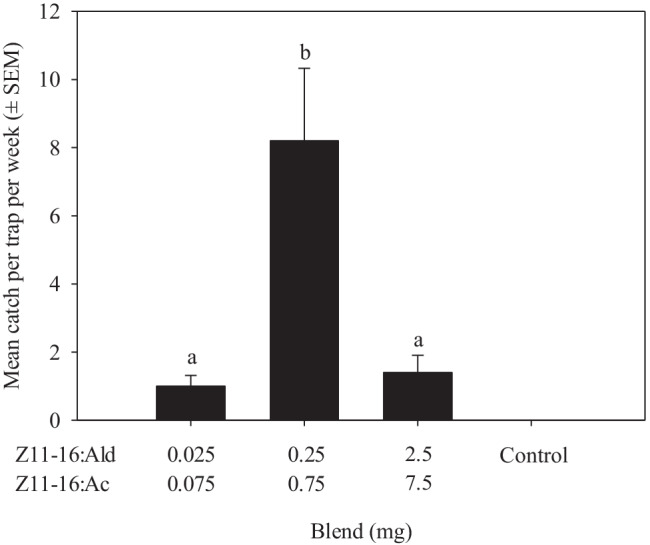
Mean catch ± SEM of *Tmetolophota atristriga* in traps baited with three doses of a binary blend of (*Z*)-11-hexadecenal (Z11-16:Ald) and (*Z*)-11-hexadecenyl acetate (Z11-16:Ac) at a ratio of 25:75*.* Different letters above columns indicate significant differences (*P* < 0.05)

The amount of the binary blend affected the number of *T. atristriga* caught (Treatment, F_2,12_ = 28.9, *P* < 0.01; Fig. 3). Increasing the dose from 0.1 to l mg resulted in an increase in catch (*P* < 0.01). Increasing the dose further to 10 mg resulted in a reduction in catch compared with the 1 mg dose (Fig. 3).

### Minor Compounds Identified in the Sex Pheromone Gland

**Fig. 4:**
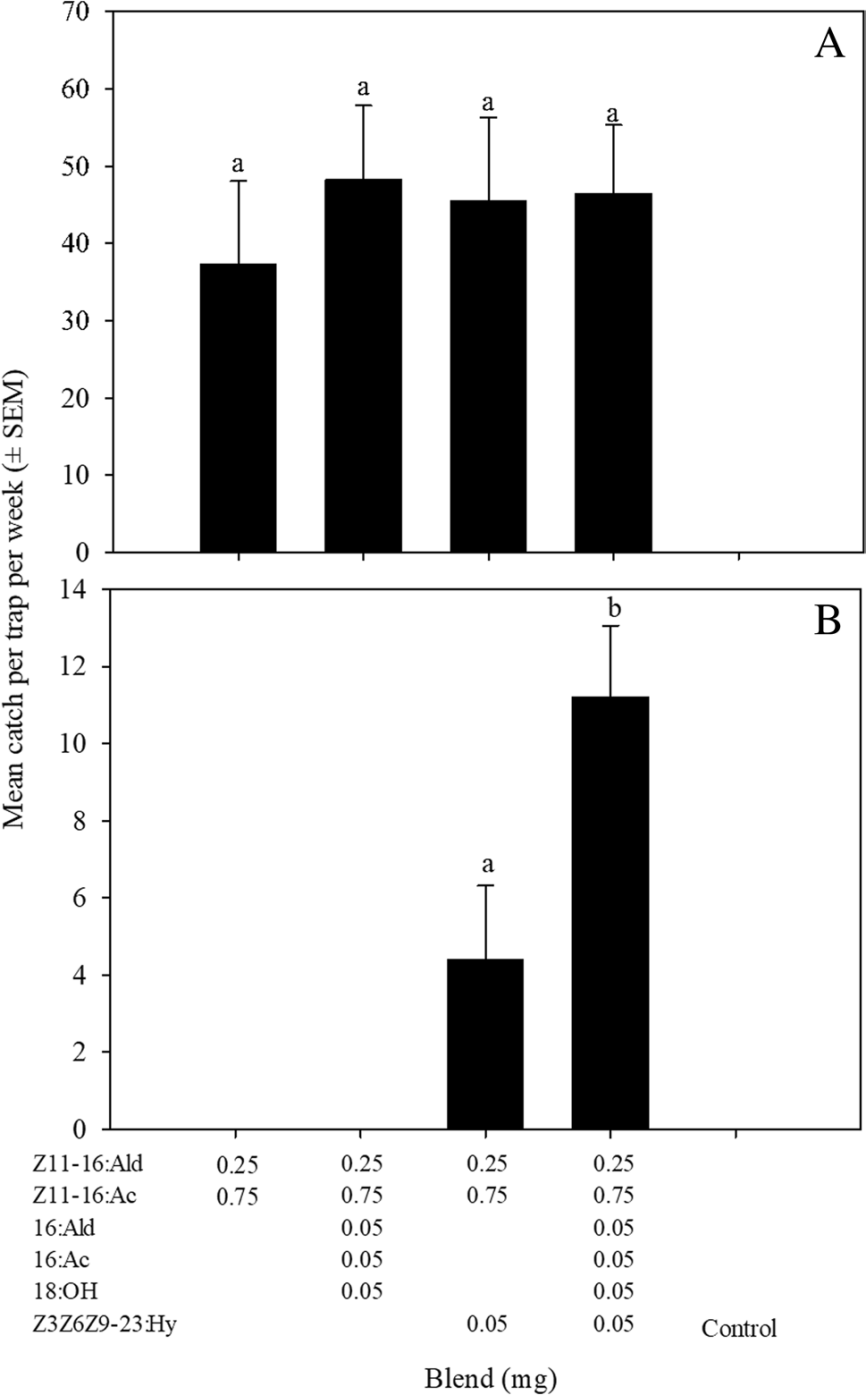
Mean catch ± SEM of *Tmetolophota atristriga* in traps baited with four blends of the six candidate pheromone compounds [(*Z*)-11-hexadecenal (Z11-16:Ald), (*E*)-11-hexadecenal, (*Z*)-11-hexadecenyl acetate (Z11-16:Ac), (*E*)-11-hexadecenyl acetate, hexadecanal (16:Ald), hexadecyl acetate (16:Ac), octadecanol (18:OH) and (3*Z*,6*Z*,9*Z*)-tricosa- 3,6,9-triene (Z3Z6Z9-23:Hy)] found in the female sex pheromone gland*.* The trial was conducted in two locations: Lincoln (A) and Little River (B). Different letters above columns indicate significant differences (*P* < 0.05)

At Lincoln, the addition of the minor components, in three combinations (three, five or six compound blends) to the binary blend did not result in an increase in the number of males caught relative to the binary blend (Treatment, F_3,16_ = 0.23, *P* = 0.87; Fig. 4A). In contrast, at Little River, no males were caught in traps baited with the binary blend (Fig. 4B). In fact, males were only caught in traps baited with blends including Z3Z6Z9-23Hy: the binary blend plus Z3Z6Z9-23Hy and the full 6-component blend. The greatest catch was obtained in traps baited with the full 6-component blend (Treatment, F_1,8_ = 6.6, *P* < 0.05; Fig. 4B).

### Dose–Response of Six-Component Blend

**Fig. 5:**
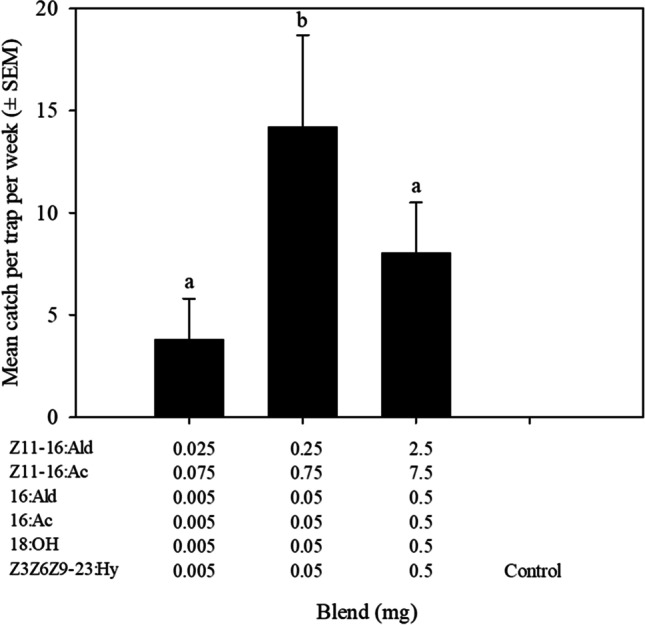
Mean catch ± SEM of *Tmetolophota atristriga* in traps baited with three doses of the six-component blend of (*Z*)-11-hexadecenal (Z11-16:Ald), (*E*)-11-hexadecenal, (*Z*)-11-hexadecenyl acetate (Z11-16:Ac), (*E*)-11-hexadecenyl acetate, hexadecanal (16:Ald), hexadecyl acetate (16:Ac), octadecanol (18:OH) and (3*Z*,6*Z*,9*Z*)-tricosa- 3,6,9-triene (Z3Z6Z9-23:Hy)*.* Different letters above columns indicate significant differences (*P* < 0.05)

The amount of the six-component blend affected the number of *T. atristriga* caught (Treatment, F_3,16_ = 4.7, *P* < 0.01; Fig. 5). Increasing the dose from 0.1 to l mg resulted in an increase in the number of males caught (*P* < 0.0001), whereas increasing the dose further to 10 mg resulted in a reduction.

## Discussion

The sex pheromone gland of female, *T. atristriga* contained at least six candidate pheromone compounds that elicited EAD responses from male antennae. None of these compounds was attractive when tested alone, but traps baited with a binary blend of Z11-16:Ac and Z11-16Ald, at a ratio of 75:25, caught males in one location (Lincoln), but not in another location (Little River) 50 km away. Addition of the minor compounds (16:Ald, 16:Ac, 18:OH and Z3Z6Z9-23:Hy) to the binary blend did not enhance catch at Lincoln but did so at Little River; addition of Z3Z6Z9-23:Hy to the binary blend resulted in catches of males, while further addition of 16:Ald, 16:Ac, and 18:OH gave an increase in male catch. Thus, of the four minor compounds, only Z3Z6Z9-23:Hy was found unequivocally to enhance trap captures when added to the binary blend. Further tests need to be carried out to find out which of 16:Ald, 16:Ac or 18:OH influence trap capture. Z3Z6Z9-23:Hy has previously been reported as a pheromone component in Crambid, Arctiid and Geometridae species (El-Sayed 2021) and now, for the first time, in a noctuid species. This adds to the growing list of lepidopteran species that use aliphatic aldehydes, alcohols or acetates in combination with polyunsaturated hydrocarbon components in their pheromone blends.

The differences in catch to the different blends at the two sites suggest there may be a difference in male response to pheromone between populations of *T. atristriga* in Canterbury, although this difference may also be explained, at least in part, by different population densities indicated by the much lower catches of males at Little River than at Lincoln. In contrast to male catch, analysis of the pheromone gland by GC/MS and GC/EAD indicated similar gland contents and male EAD-response profiles of the two populations. Therefore, even if males respond differently, it seems unlikely that there is any reproductive barrier between these two populations. This apparent difference in male response between the two sites should be tested more rigorously, using more discriminatory amounts and ratios. Differences in sex pheromone systems within a described species have been reported in other New Zealand moth species (Galbreath et al. 1985; Gibb et al. 2006; Foster et al. 1989; Frérot and Foster 1991).

Of the trapping systems tested, green bucket traps baited with 1 mg of either the binary blend of Z11-16:Ac and Z11-16:Ald at a ratio of 75:25 or the six components were effective for monitoring *T. atristriga* populations in the two locations. Although we included a Bayer dog flea collar killing strip, containing 5% Diazinon insecticide, it may be possible to refine the trapping system because *T. atristriga* males were caught in reasonable numbers in bucket traps without an insecticidal strip. Pheromone-baited traps could be useful for population suppression through mass trapping (El-Sayed et al. 2006) or lure and kill (El-Sayed et al. 2009).

A sex pheromone has been identified for only one other endemic New Zealand noctuid species, *Graphania mutans* (Walker) (Frérot and Foster 1991). Hence, our report is the second identification. The composition of the sex pheromone of *T. atristriga* shows some similarities with the sex pheromone of the Australian bollworm, *Helicoverpa punctigera* (Wallengren), with the two species having two components in common, Z11-16:Ald and Z11-16:Ac (Rothschild et al. 1982). Further sex pheromone identifications of New Zealand noctuid species will help elucidate pheromone similarities/differences with Noctuidae around the world.

## Data Availability

Data used in this study are available from the corresponding author upon request.
